# Prediction model of renal function recovery for primary membranous nephropathy with acute kidney injury

**DOI:** 10.1186/s12882-022-02882-9

**Published:** 2022-07-13

**Authors:** Tianxin Chen, Ying Zhou, Jianfen Zhu, Xinxin Chen, Jingye Pan

**Affiliations:** 1 Department of nephrology, The First Affiliated Hospital of Wenzhou Medical University, Wenzhou, China; 2 Department of endoscopy Center, The First Affiliated Hospital of Wenzhou Medical University, Wenzhou, China; 3 Department of ICU, The First Affiliated Hospital of Wenzhou Medical University, Wenzhou, 325000 Zhejiang Province PR China; 4 Key Laboratory of Intelligent Critical Care and Life Support Research of Zhejiang Province, Beijing, China

**Keywords:** Acute kidney injury, Nomogram, Renal function recovery, Membranous nephropathy, Nephrotic syndrome

## Abstract

**Background and objectives:**

The clinical and pathological impact factors for renal function recovery in acute kidney injury (AKI) on the progression of renal function in primary membranous nephropathy (PMN) with AKI patients have not yet been reported, we sought to investigate the factors that may influence renal function recovery and develop a nomogram model for predicting renal function recovery in PMN with AKI patients.

**Methods:**

Two PMN with AKI cohorts from the Nephrology Department, the First Affiliated Hospital of Wenzhou Medical University during 2012–2018 and 2019–2020 were included, i.e., a derivation cohort during 2012–2018 and a validation cohort during 2019–2020. Clinical characteristics and renal pathological features were obtained. The outcome measurement was the recovery of renal function within 12 months. Lasso regression was used for clinical and pathological features selection. Prediction model was built and nomogram was plotted. Model evaluations including calibration curves were performed.

**Result:**

Renal function recovery was found in 72 of 124 (58.1%) patients and 41 of 72 (56.9%) patients in the derivation and validation cohorts, respectively. The prognostic nomogram model included determinants of sex, age, the comorbidity of hypertensive nephropathy, the stage of glomerular basement membrane and diuretic treatment with a reasonable concordance index of 0.773 (95%CI,0.716–0.830) in the derivation cohort and 0.773 (95%CI, 0.693–0.853) in the validation cohort. Diuretic use was a significant impact factor with decrease of renal function recovery in PMN with AKI patients.

**Conclusion:**

The predictive nomogram model provides useful prognostic tool for renal function recovery in PMN patients with AKI.

**Supplementary Information:**

The online version contains supplementary material available at 10.1186/s12882-022-02882-9.

## Introduction

Acute kidney injury (AKI) is a relatively frequent complication among patients with idiopathic nephrotic syndrome (NS) [1, 2]. Primary membranous nephropathy (PMN) and minimal change disease are the two most common renal pathological types of NS. AKI has been reported to occur in 25–35% of patients with minimal change disease (MCD) [2–8]. There are few studies to investigate the epidemiology, pathophysiology and prognosis of AKI in PMN patients. Our previous study showed the incidence of AKI in PMN was similar to that in MCD. Compared with AKI in MCD, AKI in PMN was usually mild and easy to be overlooked but had lower renal function recovery rate [1, 9]. The present study builds upon our previous findings by conducting continuous follow-up of AKI patients in PMN with NS. Conducting this additional follow-up evaluation may help us to further understanding the clinical features and prognosis of these patients. The present study is exploratory in nature, with two aims. The first aim was to assess the factors that may influence renal function recovery. The second aim was to develop a nomogram model for predicting renal recovery in PMN with AKI patients.

## Patients and methods

### Patients

Between Jan 2012 to Dec 2018, a retrospective derivation cohort of PMN with AKI was established and described in our previous study [9]. The validation cohort using the same criteria was identified from the Nephrology Department, the First Affiliated Hospital of Wenzhou Medical University between Jan 2019 to Dec 2020. Baseline demographic, clinical characteristics and renal pathological changes were derived from the electronic medical system. This retrospective study protocol was approved by the ethics committees of the First Affiliated Hospital of Wenzhou Medical University.

### Data collection and follow-up

Extended follow up of PMN with AKI patients were conducted. Patients would have follow-up for at least 12 months after discharge from hospital. Except the data reported in previous study [9], the comorbidity of infection, thrombosis, diabetes and hypertensive nephropathy and laboratory data of Anti-Phospholipase A2 receptor (Anti-PLA2R) antibody were collected in this study.

### Definition and primary outcome

Hypertensive nephropathy was defined as medical history of hypertension with histological lesions of myointimal hyperplasia of arterioles, hyaline arteriosclerosis, wrinkling of basement membrane, collapse of the glomerular tuft, ischemic glomerulosclerosis and tubulointerstitial involvement. The primary outcome in this study was complete renal function recovery, which was defined as Scr convalescence to the patient’s pre-AKI baseline.

### Statistical analysis

Data was presented as mean ± SD for continuous variables and number (frequency, %) for categorical variables. Missing data was addressed by multiple imputation. Parameters were compared using the analysis of variance test or χ2 test. *P* < 0.05 was considered as significance. The least absolute shrinkage and selection operator (LASSO) analysis including 20-fold cross-validation via minimum criteria and the one standard error of the minimum criteria (the 1-SE criteria) was used to select the most useful predictive factors from the derivation cohort. The predictive factors identified by LASSO were entered into Cox regression. Variables that were statistically significant were used to construct the final model. The optimal cut-offs were chosen based on the highest Youden Index and then the nomogram model was plotted accordingly to predict the individual probability of 3-month, 6-months and 12 -month renal function recovery in PMN patients with AKI. Calibration curves were plotted to assess the performance of nomogram in the derivation and validation cohorts.

## Results

### Patients’ characteristics and the outcome of renal function recovery

72 of 124 (58.1%) AKI patients in derivation cohort and 41 of 72 (56.9%) validation cohort had complete renal function recovery. The characteristics of patients from two cohorts stratified by renal function recovery were listed in Table 1. The clinical features of patients with renal function recovery were compared with those non-recovery patients in the derivation and validation cohort. Diuretic use was significantly lower in patients with renal function recovery than that in non-recovery patients (6.9 vs 67.3% and 7.3 vs 64.5% in derivation and validation cohort respectively). Patients with renal function recovery were tended to be younger than nonrecovery patients (56 ± 12 vs 60 ± 10 and 56 ± 13 vs 62 ± 9 in derivation and validation cohort respectively). Recovery patients had lower proportion of male than nonrecovery patients (55.6 vs 82.7% and 65.9 vs 80.6% in derivation and validation cohort respectively). In validation cohort, more recovery patients received methylprednisolone (MP) with cyclophosphamide (CTX). There were no significant differences in other characters among four groups.

**Table 1:** The characteristics of derivation and validation cohort stratified by renal function recovery

Characteristics	Derivation cohort (*n* = 124)		Validation cohort (*n* = 72)		*P*
	nonrecovery (*n* = 52)	recovery (*n* = 72)	nonrecovery (*n* = 31)	recovery (*n* = 41)	
sex (male), n (%)	43 (82.7)	40 (55.6)	25 (80.6)	27 (65.9)	0.005^a^
age (yr)	60 ± 10	56 ± 12	62 ± 9	56 ± 13	0.023^b^
SBP (mmHg)	146 ± 25	141 ± 21	147 ± 23	142 ± 19	0.523
DBP (mmHg)	81 ± 12	82 ± 14	80 ± 12	82 ± 13	0.865
hgb(g/l)	125 ± 21	126 ± 17	122 ± 18	125 ± 17	0.863
glucose (mmol/l)	5.3 ± 1.4	4.9 ± 1.1	4.9 ± 1.2	5.0 ± 1.3	0.383
TC (mmol/l)	8.3 ± 2.4	8.2 ± 2.9	8.0 ± 2.0	7.9 ± 2.9	0.860
TG (mmol/l)	3.3 ± 3.0	3.1 ± 2.1	2.9 ± 1.7	3.2 ± 2.5	0.875
HDL (mmol/l)	1.3 ± 0.4	1.4 ± 1.1	1,3 ± 0.4	1.3 ± 0.6	0.689
LDL (mmol/l)	5.0 ± 2.2	4.8 ± 2.2	4.8 ± 1.7	4.5 ± 2.1	0.660
Scr_max_ (umol/l)	121 ± 32	111 ± 27	122 ± 34	111 ± 20	0.076
Salb (gl/l)	19.6 ± 3.9	20.7 ± 3.5	19.7 ± 3.7	20.7 ± 3.9	0.239
Upro (g/24 h)	7.3 ± 4.0	6.7 ± 3.3	6.4 ± 2.8	6.9 ± 3.6	0.722
Anti-PLA2R positive，n(%)	49 (94.2)	70 (97.2)	30 (96.8)	41 (100)	0.457
AKI stage, n (%)					
1 stage	37 (71.2)	63 (87.5)	22 (71.0)	39 (95.1)	0.005^c^
2 stage	13 (25.0)	8 (11.1)	8 (25.8)	1 (2.4)	0.006^d^
3 stage	2 (3.8)	1 (1.4)	1 (3.2)	1 (2.4)	0.849
GBM stage, n (%)					
I stage	40 (76.9)	50 (69.4)	22 (71.0)	30 (73.2)	0.827
II stage	12 (23.1)	22 (30.6)	9 (29.0)	11 (26.8)	0.827
Infection, n (%)	4 (7.7)	5 (6.9)	3 (9.7)	2 (4.9)	0.886
Thrombosis, n (%)	0 (0)	1 (1.4)	0 (0)	1 (2.4)	0.622
Hypertensive nephropathy, n(%)	41 (78.8)	59 (81.9)	24 (77.4)	33 (80.1)	0.951
Diabetes, n (%)	12 (23.1)	16 (22.2)	7 (29.3)	12 (24.0)	0.848
MPCTX, n (%)	23 (44.2)	44 (61.1)	9 (29.0)	25 (61.0)	0.009^e^
FK506, n (%)	17 (32.7)	23 (31.9)	10 (32.3)	13 (31.7)	1.000
CSA, n (%)	7 (13.5)	13 (18.1)	4 (12.9)	3 (7.3)	0.462
RSAI, n (%)	48 (92.3)	64 (88.9)	30 (96.8)	37 (90.2)	0.609
Diuretics, n (%)	35 (67.3)	5 (6.9)	20 (64.5)	3 (7.3)	< 0.001^f^

*SBP* Systolic blood pressure, *DBP* Diastolic blood pressure, *hbg* Hemoglobin, TC Total cholesterol, *TG* Triglycerides, *HDL* High-density lipoprotein, *LDL* Low-density lipoprotein, *Salb* Serum albumin, *Upro* Urine protein, *GBM* Glomerular basement membrane, *MPCTX* Methylprednisolone with cyclophosphamide, *FK506* Tacrolimus, *CsA* Cyclosporin A, *RSAI* Renin angiotesin system inhibitors

^a^Derivation cohort: non-recovery vs recovery, *p* = 0.022; Validation cohort: non-recovery vs recovery, *p* = 0.004;

^b^Derivation cohort: non-recovery vs recovery, *p* = 0.041; Validation cohort: non-recovery vs recovery, *p* = 0.024;

^c^Derivation cohort: non-recovery vs recovery, *p* = 0.022; Validation cohort: non-recovery vs recovery, *p* = 0.004

^d^Derivation cohort: non-recovery vs recovery, *p* = 0.042; Validation cohort: non-recovery vs recovery, *p* = 0.003

^e^Derivation cohort: non-recovery vs recovery, *p* = 0.063; Validation cohort: non-recovery vs recovery, *p* = 0.007

^f^Derivation cohort: non-recovery vs recovery, *p* < 0.001; Validation cohort: non-recovery vs recovery, *p* < 0.001

### Feature selection, nomogram model and calibration curves

**Fig. 1:**
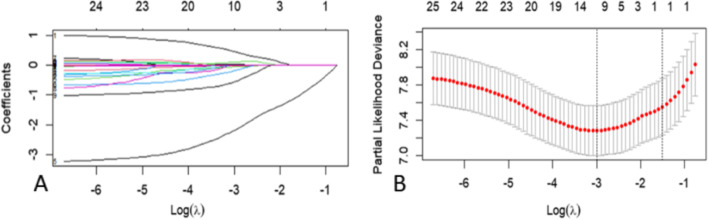
Feature selection by the least absolute shrinkage and selection operator (LASSO) model. **A** LASSO coefficient profiles of 25 clinical and pathological features. **B** The deviance profiles of LASSO Cross-Validation

**Fig. 2:**
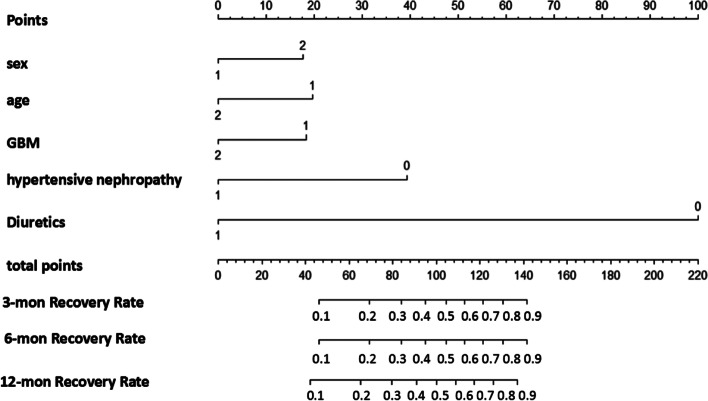
Nomogram of prediction model based on derivation. Sex: 1. male, 2. female; age:1. < 55 yr, 2. > 55 yr; GBM: 1. stage I, 2. stage II; hypertensive nephropathy: 1. patients with comorbidity of hypertensive nephropathy, 0. without hypertensive nephropathy; Diuretics: 1. patients with diuretic use, 0. without diuretic use

10 potential determinants (Supplemental Table 1) were selected from 25 clinical and pathological features (treatment included) based on the derivation cohort by LASSO regression model (Fig. 1). The optimal cut-off of age was set by ROC analysis (Supplementary Table 2). Cox logistic regression was further performed to confirm that 5 determinants were independent clinical predictors for renal function recovery (Table 2). With sex, age, glomerular basement membrane (GBM) stage, hypertensive nephropathy and diuretic use, the proposed nomogram model was developed with C-index of 0.773 (95%CI, 0.716–0.830) in the derivation cohort and 0.773 (95%CI, 0.693–0.853) in the validation cohort, respectively. Nomogram of prediction model was plotted in Fig. 2. The predicted recovery probability of renal function during 3-month, 6-month and 12-month can be determined in nomogram models. Each variable was given a score on the points scale. If a 60-year-old female PMN with AKI patient has stage-1 GBM pathological change, the corresponding score is approximately 35. If the patient has the comorbidity of hypertensive nephropathy and does not require diuretic treatment, the corresponding score is approximately 100. In this case, by adding up the total score was 135, which indicates the 3-, 6- and 12-month recovery rates would be 0.7, 0.7 and 0.75 respectively.

**Table 2:** Five determinants for predicting recovery base on Cox regression

Impact Factor	Exp(B)	95.0% CI		*P*
Age (> 55 yr)	0.535	0.33	0.867	0.011
Sex (female)	1.745	1.091	2.791	0.02
GBM (II stage)	0.560	0.329	0.953	0.033
Hypertensive nephropathy	0.294	0.145	0.595	0.001
Diuretics	0.047	0.014	0.119	< 0.001

*GBM* Glomerular basement membrane

**Fig. 3:**
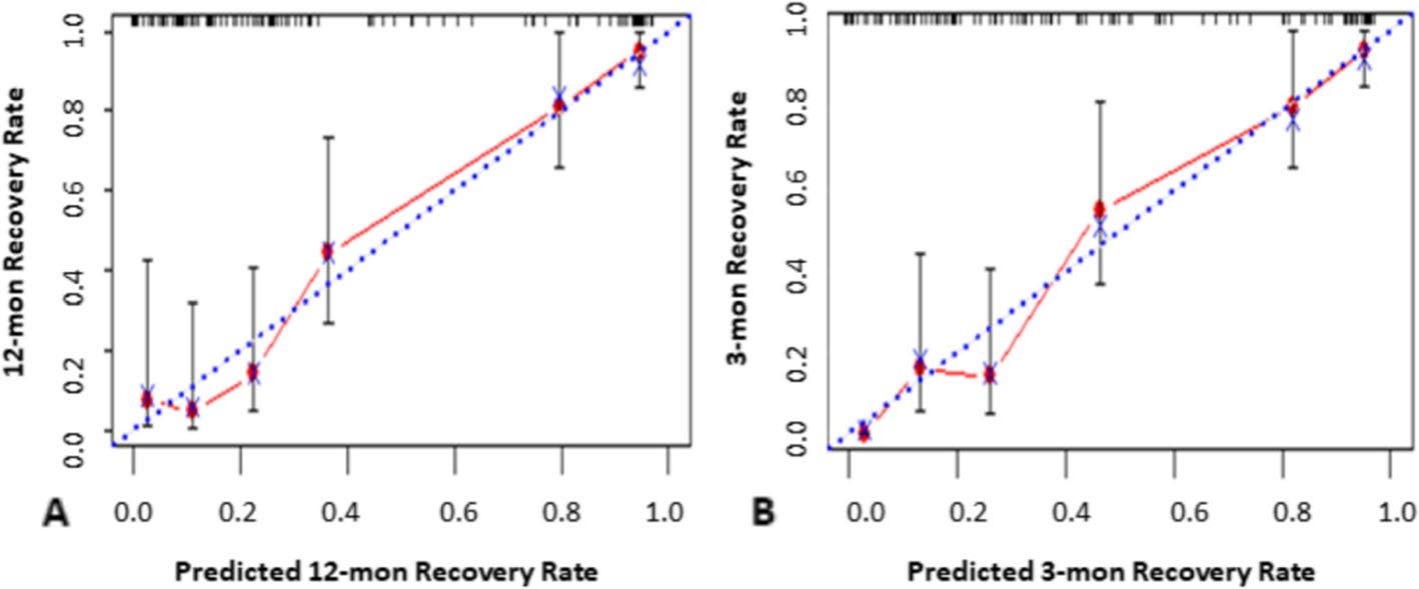
The calibration curves of the nomogram Model in derivation cohort. **A** predictive recovery probability and observational recovery probability during 12 months; **B** predictive recovery probability and observational recovery probability during 3 months

**Fig. 4:**
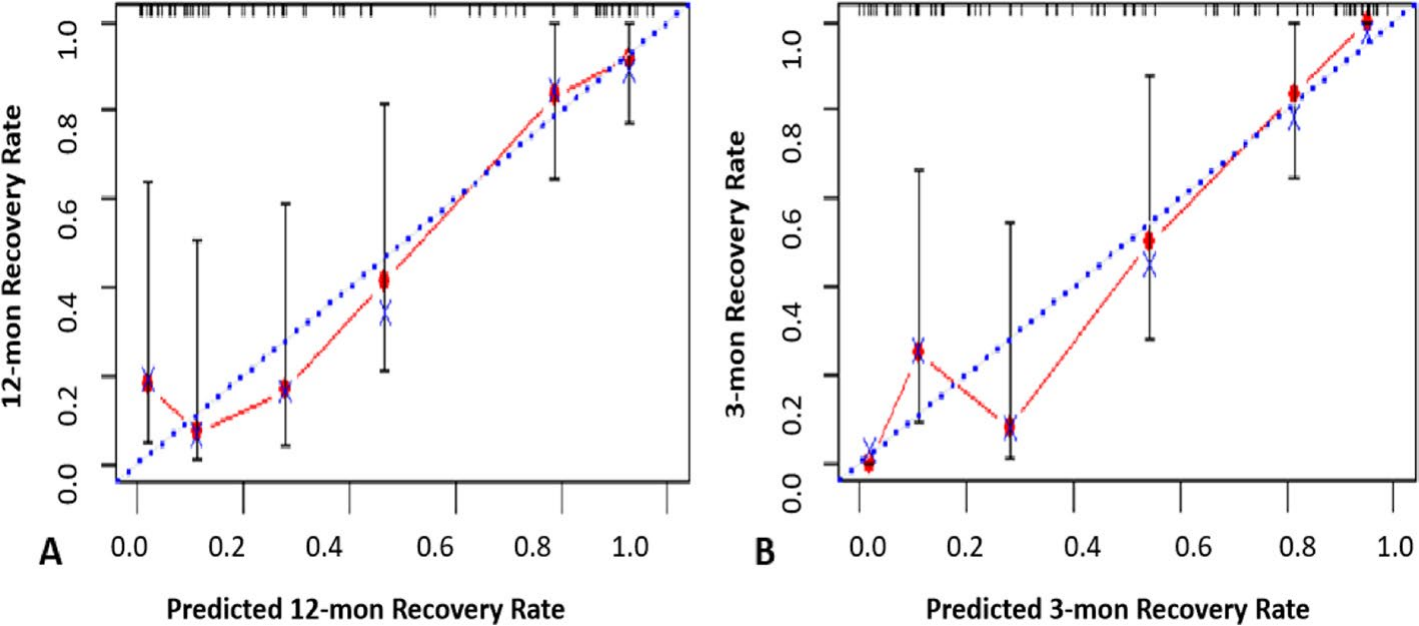
The calibration curves of the nomogram Model in validation cohort. A, predictive recovery probability and observational recovery probability during 12 months; B, predictive recovery probability and observational recovery probability during 3 months

The calibration curves of the nomogram Model to predict the renal function recovery during 3 months and 12 months after AKI, demonstrated good consistency between predictive recovery probability and observational recovery probability in both derivation (Fig. 3) and validation cohort (Fig. 4).

## Discussion

Most previous retrospective studies aimed to investigate the incidence, risk factors, and the associated outcomes of AKI in NS [1–4]. To date, few reports have discussed on the impact factors of renal function recovery in PMN with AKI. The published literature has been limited to the retrospective analysis of risk factors associated with the development of AKI in NS. In this study, we sought to identify specific factors affecting the renal function recovery of PMN with AKI patients. The outcome of AKI in PMN patients is very complicated and multifactorial in origin. To evaluate this, 25 variables including clinical characters, pathological changes, comorbidities and treatments were plotted. Ten potential determinants were selected from the derivation cohort by LASSO regression model and multivariate Cox regression analysis confirmed that male gender, age(> 55 yr), GBM (II stage), hypertensive nephropathy and diuretic use were important impact factors for renal function recovery. A nomogram model for predicting 3-month, 6-month and 12-month outcomes of AKI in PMN was developed and furthermore we validated the model of power parallel speedup through simulation and calibration analysis.

Although diuretics are used commonly in AKI, there is no clear evidence that they improve outcomes in AKI. In our study, more than 60% nonrecovery patients were administrated with diuretics and diuretic use was associated with a significant decrease of renal function recovery (odds ratio, 0.047; 95%CI, 0.014–0.119) in PMN with AKI patients. According to the study reported by Mehta et al., diuretic use was associated with an increased in-hospital mortality and nonrecovery of renal function in critically ill patients with acute renal failure [10]. A recent study using real-world data reported that diuretics (furosemide) administration was associated with improved recovery of renal function in critically ill patients with AKI but it was not effective in those with chronic kidney disease [11]. Randomized blinded controlled trials showed furosemide did not improve renal function recovery in critical ill patients [12, 13].

Our study found one important clinical implication that hypertensive nephropathy was the risk factor for nonrecovery of renal function in PMN with AKI. It is well known that hypertensive nephropathy is second only to diabetes as a leading cause of progressive chronic kidney disease [14]. Major aspects of clinical hypertensive renal damage remain poorly understood [15]. Dysfunction of renal autoregulation due to myointimal hyperplasia of arterioles and hyaline arteriosclerosis was recognized to contribute significantly to the deterioration of renal function. In recent years, novel evidence has demonstrated that persistent high blood pressure injures tubular cells, leading to epithelial-mesenchymal transition and changes in post-glomerular peritubular capillaries induce endothelial damage and hypoxia [16]. Microvasculature dysfunction by inducing hypoxic environment may be the main pathophysiological mechanisms mediating poor functional recovery in AKI accompanied by hypertensive nephropathy.

Rates of renal function recovery from AKI differ dramatically among populations and can vary between 33 and 90% in published studies [17–25]. Recovery rates of AKI patients in our study were 58.1 and 56.9% in derivation cohort and validation cohort respectively. In derivation cohort, our previous study showed 16(12.9%) of 124 AKI patients progressed slowly and were diagnosed as chronic renal insufficiency. However, 6 of these 16 patients had complete recovery of renal function for more than 3 months after diagnosis in the present extended follow up study.

There are several limitations of our study. First and for most, it was a retrospective observational study with limited sample size from a single center. Although the predictive model calibrated in validation cohort, prospective multi-center studies are mandatory to further validate the utility of our model. Second, there are no adult guidelines available on managing oedema and volume overload in nephrotic syndrome. The lack of guidelines means that there is considerable heterogeneity in the treatment of overloaded nephrotic individuals. There was also no consensus on the indication, starting dose, dosage change and monitoring of fluid balance; consequently, there are considerable differences in treatment pathways in our study. The association of diuretic type and dosage with renal recovery was not deeply analyzed. Third, extensive pathohistological data mining along with emerging biomarkers will probably offer more detailed information for the prediction of recovery, but was not plotted in this study. Fourth, Corticorsteriods with CTX or calcineurin inhibitors were used as initial therapy based on KDIGO guidelines. In order to reduce the risk of toxicity, the doses of cyclophosphamide or calcineurin inhibitors were adjusted according to patients’age and estimated glomerular filtration rate. Immunosuppressive induction agents were not mutually exclusive (e.g. some patients would be prescribed with low-dose corticosteroids with calcineurin inhibitors after first cycle of high-dose corticosteroids with CTX regimens due to significant adverse effects), which may be the main reason that induction agents was not the impact factor for recovery in our study.

In conclusion, our two-sets of nomogram provides useful prognostic tool for renal function recovery with in PMN patients with AKI. The prognostic model assists clinicians’ decision making, for instances, to facilitate timely appropriate treatments to void nephrotoxic drugs and also to tailor the diuretic treatment for PMN patients with delayed renal function recovery.

## Supplementary Information

**Additional file 1: Supplemental Table 1.** The coefficients of 10 potential determinants by LASSO regression. **Supplemental Table 2.** The optimal cut-off of age determined by ROC analysis.

## Data Availability

The data are available from the first author upon reasonable request and with permission of the First Affiliated Hospital of Wenzhou Medical University.

## Abbreviations

AKI: Acute kidney injury
PMN: Primary membranous nephropathy
NS: Nephrotic syndrome
MCD: Minimal change disease
Anti-PLA2R: Anti-Phospholipase A2 receptor antibody
LASSO: The least absolute shrinkage and selection operator
MP: Methylprednisolone
CTX: Cyclophosphamide
GBM: Glomerular basement membrane
KDIGO: Kidney Disease: Improving Global Outcomes
