# Supplementary material for: Prediction model of renal function recovery for primary membranous nephropathy with acute kidney injury
Source: BMC Nephrol. 2022 Jul 13;23:247. doi: 10.1186/s12882-022-02882-9 (PMC9281044; doi:10.1186/s12882-022-02882-9)
Supplement: Supplementary file 1 — Additional file 1: Supplemental Table 1. The coefficients of 10 potential determinants by LASSO regression. Supplemental Table 2. The optimal cut-off of age determined by ROC analysis. [file 12882_2022_2882_MOESM1_ESM.docx]

**Supplemental tables**

Supplemental table 1. The coefficients of 10 potential determinants by LASSO regression.

Supplemental table 2. The optimal cut-off of age determined by ROC analysis.

Supplemental table 1. The coefficients of 10 potential determinants by LASSO regression.

| Potential determinants | coefficients |
| --- | --- |
| sex | 0.408 |
| age | -0.016 |
| TG | -0.003 |
| Upro | 0.021 |
| PLA2R | 0.000 |
| AKIstage | -0.054 |
| GBM | -0.141 |
| hypertensive nephropathy | -0.500 |
| MPCTX | 0.112 |
| Diuretics | -2.107 |

TG, triglycerides; Upro, urine protein; GBM, glomerular basement membrane; MPCTX, methylprednisolone with cyclophosphamide.

Supplementary table 2. The optimal cut-off of age determined by ROC analysis

| age | sensitivity | 1-specificity | Youden Index |
| --- | --- | --- | --- |
| 22 | 1 | 1 | 0 |
| 25 | 1 | 0.981 | 0.019 |
| 27.5 | 0.986 | 0.981 | 0.005 |
| 28.5 | 0.972 | 0.981 | -0.009 |
| 29.5 | 0.958 | 0.981 | -0.023 |
| 32 | 0.944 | 0.981 | -0.037 |
| 35.5 | 0.931 | 0.981 | -0.05 |
| 37.5 | 0.917 | 0.981 | -0.064 |
| 38.5 | 0.903 | 0.981 | -0.078 |
| 40 | 0.875 | 0.981 | -0.106 |
| 41.5 | 0.861 | 0.962 | -0.101 |
| 42.5 | 0.861 | 0.942 | -0.081 |
| 43.5 | 0.833 | 0.942 | -0.109 |
| 44.5 | 0.819 | 0.942 | -0.123 |
| 45.5 | 0.792 | 0.923 | -0.131 |
| 46.5 | 0.764 | 0.865 | -0.101 |
| 47.5 | 0.75 | 0.865 | -0.115 |
| 48.5 | 0.722 | 0.846 | -0.124 |
| 50 | 0.694 | 0.827 | -0.133 |
| 51.5 | 0.667 | 0.808 | -0.141 |
| 52.5 | 0.639 | 0.808 | -0.169 |
| 53.5 | 0.611 | 0.75 | -0.139 |
| **55** | **0.542** | **0.75** | **-0.208** |
| 56.5 | 0.514 | 0.712 | -0.198 |
| 57.5 | 0.514 | 0.692 | -0.178 |
| 58.5 | 0.486 | 0.654 | -0.168 |
| 59.5 | 0.472 | 0.596 | -0.124 |
| 60.5 | 0.431 | 0.481 | -0.05 |
| 61.5 | 0.389 | 0.442 | -0.053 |
| 62.5 | 0.375 | 0.423 | -0.048 |
| 63.5 | 0.333 | 0.404 | -0.071 |
| 64.5 | 0.236 | 0.404 | -0.168 |
| 65.5 | 0.194 | 0.308 | -0.114 |
| 66.5 | 0.194 | 0.25 | -0.056 |
| 67.5 | 0.181 | 0.25 | -0.069 |
| 68.5 | 0.153 | 0.212 | -0.059 |
| 69.5 | 0.111 | 0.192 | -0.081 |
| 70.5 | 0.097 | 0.154 | -0.057 |
| 71.5 | 0.083 | 0.115 | -0.032 |
| 72.5 | 0.056 | 0.077 | -0.021 |
| 73.5 | 0.028 | 0.058 | -0.03 |
| 74.5 | 0.014 | 0.058 | -0.044 |
| 75.5 | 0.014 | 0.038 | -0.024 |
| 76.5 | 0 | 0.019 | -0.019 |
| 78 | 0 | 0 | 0 |

Optimal cut-off of age was 55 years when Youden Index is -0.208.
